# Muscle Transcriptome Analysis Reveals Potential Candidate Genes and Pathways Affecting Intramuscular Fat Content in Pigs

**DOI:** 10.3389/fgene.2020.00877

**Published:** 2020-08-11

**Authors:** Xueyan Zhao, Hongmei Hu, Haichao Lin, Cheng Wang, Yanping Wang, Jiying Wang

**Affiliations:** Shandong Provincial Key Laboratory of Animal Disease Control and Breeding, Institute of Animal Science and Veterinary Medicine, Shandong Academy of Agricultural Sciences, Jinan, China

**Keywords:** pork quality, IMF content, RNA sequencing, differentially expressed genes, WGCNA

## Abstract

Intramuscular fat (IMF) content plays an essential role in meat quality. For identifying potential candidate genes and pathways regulating IMF content, the IMF content and the *longissimus dorsi* transcriptomes of 28 purebred Duroc pigs were measured. As a result, the transcriptome analysis of four high- and four low-IMF individuals revealed a total of 309 differentially expressed genes (DEGs) using edgeR and DESeq2 (*p* < 0.05, |log_2_(fold change)| ≥ 1). Functional enrichment analysis of the DEGs revealed 19 hub genes significantly enriched in the Gene Ontology (GO) terms and pathways (*q* < 0.05) related to lipid metabolism and fat cell differentiation. The weighted gene coexpression network analysis (WGCNA) of the 28 pigs identified the most relevant module with 43 hub genes. The combined results of DEGs, WGCNA, and protein–protein interactions revealed *ADIPOQ*, *PPARG*, *LIPE*, *CIDEC*, *PLIN1*, *CIDEA*, and *FABP4* to be potential candidate genes affecting IMF. Furthermore, the regulation of lipolysis in adipocytes and the peroxisome proliferator-activated receptor (PPAR) signaling pathway were significantly enriched for both the DEGs and genes in the most relevant module. Some DEGs and pathways detected in our study play essential roles and are potential candidate genes and pathways that affect IMF content in pigs. This study provides crucial information for understanding the molecular mechanism of IMF content and would be helpful in improving pork quality.

## Introduction

Meat quality is the main economic trait in pig production and can be evaluated by multiple indicators, such as intramuscular fat (IMF) content, pH, water holding capacity, color, and tenderness. Of these, IMF content is arguably the most important and is closely correlated to other meat quality traits, such as flavor, juiciness, and tenderness (Klont et al., 1998). High levels of IMF content or marbling have a positive influence on the eating quality of pork (Van Laack et al., 2001). However, for the past century, thinner backfat was considered as an important parameter in pig breeding. Although this selection led to higher muscularity and growth, IMF content, juiciness, and tenderness of the meat decreased (Hernández-Sánchez et al., 2013). It is extremely difficult to improve IMF content by traditional breeding methods, even as consumers have become more discerning about meat quality. Therefore, it is worthwhile to understand the molecular basis of IMF content and carry out molecular breeding for satisfying consumer preferences.

IMF content is a complex trait, which is regulated by many genes. Over the past two decades, using low-density microsatellite markers, quantitative trait loci (QTL) linkage analyses have been performed to determine the QTL underlying IMF content in pigs (Grindflek et al., 2001; Ma et al., 2009). However, due to the low density of markers, these QTL represent large chromosomal regions, and it is difficult for further fine mapping to identify causative genes (Pearson and Manolio, 2008). The emergence of high-throughput genotyping platforms, such as single nucleotide polymorphism (SNP) arrays, makes it possible to find genetic variants and QTL associated with traits of interest in a narrower region. Genome-wide association studies (GWAS), using pig-specific high-density SNP chips, have made substantial progress in identifying genetic factors associated with or underlying IMF content (Roger et al., 2016; Wang et al., 2020). To date, 709 QTL for IMF content have been deposited in the pigQTLdb (Release 41, Apr 26, 2020)^1^. However, when compared with other traits, the progress of dissecting the genetic basis for IMF content is still limited due to its complexity.

RNA sequencing (RNA-seq), a method based on next-generation sequencing (NGS), offers opportunities to provide unprecedented details about the transcriptional landscape of a certain organism. Compared with real-time PCR and microarrays, RNA-seq, with low background noise and high dynamic ranges of gene expression level quantification, integrates advanced molecular biology techniques and bioinformatics (Wang et al., 2009). Therefore, it is a powerful method for studying complex quantitative traits controlled by many interacting genes. Several studies on transcriptome profiling of porcine *longissimus dorsi* (LD) have been performed to investigate genes or pathways influencing IMF content in pigs. However, most of these studies have been carried out using pigs of different breeds (Li et al., 2016, 2020; Xu J. et al., 2018), with few studies on individuals of the same breed (Lim et al., 2017; Muñoz et al., 2018) with distinct IMF content to identify consistent candidate genes. Hence, based on the RNA-seq, further studies using multiple analysis methods should be conducted to unravel genes and the pathways that regulate IMF contents.

Comparing the gene expression profile across phenotypes of interest to identify differentially expressed genes (DEGs) is the most commonly used approach in RNA-seq. Complementary to it, weighted gene coexpression network analysis (WGCNA) method, developed by Langfelder and Horvath (2008) in the form of an R package, identifies gene sets that work cooperatively in related pathways and contribute to resulting phenotypes. To date, WGCNA has been widely used in the investigation of complex diseases and traits in humans and mice based on RNA-seq (Darlington et al., 2013; Li et al., 2015; Jin et al., 2019; Shi et al., 2020). In pigs, WGCNA was also employed to analyze a number of complex traits, such as feed efficiency (Xu Y. et al., 2018; Banerjee et al., 2020; Carmelo et al., 2020), heat tolerance (He et al., 2020), residual feed intake (Liu H. et al., 2016), and obesity (Kogelman et al., 2014). However, so far, only one study was found that have used WGCNA to analyze IMF content in Italian Large White pigs (Zappaterra et al., 2020).

The Duroc pig population is extensively used as the terminal male parent in the breeding of Duroc × Landrace × Yorkshire (DLY) commercial pigs due to its high meat quality and large muscle mass. Previous studies have shown that the Duroc breed has substantially higher levels of IMF content than other commercial breeds (Cisneros et al., 2010). Moreover, in this study, we found that IMF content of Duroc pigs also varied significantly between individuals (from 1.17 to 4.23%). Thus, individuals with extreme IMF content in the Duroc population are good specimens for transcriptomics study of IMF content. Hence, in the present study, we performed RNA sequencing of LD muscles from 28 Duroc pigs with variant IMF content, analyzed the transcriptome differences between the groups with extremely high- and low-IMF content, conducted WGCNA of all individuals, and determined key DEGs and biological pathways affecting IMF content. This study provides valuable information for understanding the molecular basis underlying IMF content in pigs.

## Materials and Methods

### Animals and Sample Collection

In this study, we selected 28 Duroc pigs (13 male and 15 female) of similar ages with an average body weight of 108.29 ± 6.00 kg (mean ± standard deviation) (Supplementary Table S1). These pigs were reared together in the same breeding farm in the Shandong province of China. The individuals were derived from different sires and dams, and thus, no sibling and half-sibling relationships existed among them. These pigs were fed on diets formulated according to their age and were provided free access to water under the same environment. They were slaughtered in one batch following stunning by electric shock, which is a common abattoir practice. All the experimental procedures were approved by the Institutional Animal Care and Use Committee of Institute of Animal Husbandry and Veterinary Medicine, Shandong Academy of Agricultural Sciences (approval code, IACC20060101, 1 January 2006). About 0.2 g of LD muscle from the last fourth and fifth thoracic vertebrae was collected for each pig, placed into a tube with RNAlater Stabilization Solution (Thermo Fisher Scientific, Waltham, MA, United States), and frozen at −80°C for RNA extraction.

For the determination of IMF content, about 200 g of LD muscle was also obtained from the last fourth and fifth thoracic vertebra of each pig. After removing the adipose and connective tissues, these muscle samples were oven dried to constant weight for removing moisture. After the samples were ground, IMF content was examined using the Soxhlet petroleum–ether method and expressed as the weight percentage of wet muscle tissue. Each sample was measured in triplicate to ensure its accuracy.

### RNA Isolation, Library Construction, and Sequencing

Total RNA was isolated from 28 porcine LD samples using the TRIzol reagent (Invitrogen, Life Technologies). To evaluate the quality of RNA, RNA degradation and contamination were first monitored on 1% agarose gels. Then, the purity and concentration of RNA were examined with NanoPhotometer^®^ spectrophotometer (IMPLEN, CA, United States) and Qubit^®^ RNA Assay Kit in Qubit^®^ 2.0 Fluorometer (Life Technologies, CA, United States), respectively. Finally, RNA integrity was confirmed using the RNA Nano 6000 Assay Kit of the Bioanalyzer 2100 (Agilent Technologies, CA, United States). RNA integrity numbers (RINs) of the samples were 8.0–8.6. A total amount of 3 μg RNA with RIN > 8.0 was used as input material for the RNA sample preparations. RNA libraries were constructed using the NEBNext^®^ Ultra^TM^ RNA Library Prep Kit for Illumina^®^ (NEB, United States) following the manufacturer’s recommendations. The method used for library construction is based on the poly-T oligo-attached magnetic beads. Messenger RNA (mRNA), including coding RNA and a small group of long non-coding RNA (lncRNA) with ploy A tail, were isolated from the total RNA. Then, the library preparations were sequenced with 15 cycles on an Illumina Hiseq platform, and 150 bp paired-end reads were generated.

### Quality Assessment of Sequencing Data and Mapping of mRNA-Seq Reads

To ensure the quality of bioinformatics analysis, raw reads were first filtered to obtain clean reads. We filtered raw reads in the FASTQ format by removing reads according to the following criteria: containing adapter sequences, ambiguous base content > 0.1%, and 50% of bases whose Q_phred_ quality score ≤ 20. Clean reads were then mapped to the *Sus scrofa* reference genome (Sscrofa11.1) and the annotation database Ensembl Genes 95 using HISAT2 software (Daehwan et al., 2015). FeatureCounts tool of subread package was used for calculating the number of reads mapped to each gene for estimating gene expression levels (Yang et al., 2014). Gene expression levels were normalized using the expected number of fragments per kilobase of exon per million fragments (FPKM).

### Differentially Expressed Genes’ Detection and Functional Enrichment Analysis

Based on the phenotypic data of IMF and sex from 28 Duroc pigs, samples from four high- (two male and two female) and four low-IMF content pigs (two male and two female) were chosen for transcriptome difference analysis. The differential expression analysis was carried out using the R package DESeq2 (Love et al., 2014) and edgeR (Robinson et al., 2010), and adjusted *p*-values (*q*-value) were calculated using Benjamini and Hochberg’s (BH) approach for controlling the false discovery rate. Compared to edgeR, DESeq2 allowing more general data gives an advantage selection of differential genes expression during the dynamic range of data (Varet et al., 2016). However, Alshehri and Alkharouf found that EdgeR had a large number of unique differential expression genes that were not shared with other tools including DESeq2 (Alshehri and Alkharouf, 2018). Hence, in the present study, overlapped DEGs were detected by edgeR and DESeq2.

Overlapped DEGs, which were detected by DESeq2 and edgeR methods in terms of |log_2_(fold change)| ≥ 1 and *p* < 0.05, were subjected to GO and Kyoto Encyclopedia of Genes and Genomes (KEGG) pathway enrichment analysis using R package clusterProfiler (Yu et al., 2012). The GO terms and pathways with *q* value (adjusted *p*-value by BH method) < 0.05 were considered to be significantly enriched ones. Genes involved in the significantly enriched GO terms and pathways related to IMF content were treated as hub genes, and more attention was paid to these in downstream WGCNA (Langfelder and Horvath, 2008).

### Weighted Gene Coexpression Network Analysis

To construct a coexpression network, we further applied WGCNA using FPKM values obtained from the mRNA-Seq of 28 Duroc pigs. The WGCNA, a comprehensive collection of R functions, was used for performing various aspects of weighted correlation network analysis (Langfelder and Horvath, 2008). Genes with FPKM values > 1 in more than 14 individuals were selected for a coexpression network setting, resulting in 10,676 genes. Based on these genes, hierarchical cluster analysis of all samples was carried out by group average method to detect outliers with a cutoff at a height of 2,000. Outliers are defined as the samples that appear to deviate markedly from other samples. After filtering the outliers, the other samples were employed for establishing an unsigned coexpression network.

Specifically, adjacency matrix *a* was first calculated by formula *a*_*ij*_ = |*s*_*ij*_|*^β^*, where s*_*ij*_* is the absolute value of the correlation coefficient between gene *i* and gene *j*, and β is a soft-power threshold. In the present study, the power of 14 based on the scale-free topology criterion was used, resulting in a scale-free topology index (*R*^2^) of 0.85. Next, to make networks less sensitive to spurious connections or to connection missing due to random noises, the topological overlap matrix (TOM) (Zhang and Horvath, 2005), representing the overlap in shared neighbors, was introduced to identify modules of highly coexpressed genes based on the adjacency matrix. The dissimilarity TOM, which was calculated by 1 minus the TOM, was used as input for the dendrogram. By hierarchical clustering and dynamic tree cutting (Langfelder et al., 2007), genes clustered into distinct modules were assigned to a color. The hybrid dynamic tree cutting method was used to cut branches using a minimum module size of 30, which is the default and commonly used value (Maertens et al., 2018). Furthermore, module eigengene (ME) (Zhao et al., 2010), representing the module expressions of each module, was calculated by the first principal component of the expression matrix. The ME can be considered as a weighted average expression profile. To identify biologically significant modules and to select potential critical modules for downstream analysis, the WGCNA approach defines module–trait relationships (MTRs) (Kogelman et al., 2014) and gene significance (GS) of each module (Liu J. et al., 2016). The Pearson’s correlations between the ME and the IMF content values and between the expression profiles and the IMF content values were calculated for estimating the MTRs and GS, respectively. Module GS (MS) was the mean value of GS of the module genes. According to the selection criteria for critical modules reported in previous studies, modules with MTRs > 0.30 and MS > 0.25 were considered as candidate modules for the following functional enrichment analysis (Howard et al., 2017; Yang et al., 2018).

Functional annotation using the R package clusterProfiler was conducted on the genes in each candidate module. The GO terms and pathways with *q* < 0.05 (adjusted *p*-value by BH method) were considered as significant. Among the candidate modules, modules were considered as critical ones when the module genes were involved in IMF-related GO terms and pathways. Meanwhile, to further identify biologically significant gene in each critical module, module membership (MM) (Howard et al., 2017) and connectivity of genes were also detected; MMs, the correlation coefficient between the gene expression and ME for module membership, were calculated to measure the gene-module membership. Intramodular connectivity of one gene, which was the sum of the adjacency matrix α between that gene and all the other genes in the module, was also calculated. Genes with GS > 0.3, MM > 0.85, and an intramodular connectivity > 5 were considered hub genes.

## Results and Discussion

### Summary of Sequencing Data and Sample Selection for Comparative Gene Expression Analysis

In this study, we measured IMF content of 28 Duroc pigs selected from a breeding farm. IMF content varied significantly among individuals, ranging from 1.17 to 4.23% with an average of 2.36% (Supplementary Table S1). The LD muscles of these 28 pigs were sequenced using a paired-end mRNA-seq approach. In total, 52.43–86.93 million raw reads per sample were generated. After filtering 2.31% of raw reads, an average of 62.56 million clean reads were used for the following analyses (Supplementary Table S2). Of these, 94.36% of clean reads were successfully mapped to the *S. scrofa* (Sscrofa11.1) genome assembly, with 90.34% being uniquely mapped (Supplementary Table S2). In addition, out of the reads mapped to the reference genome, 91.58 and 3.85% were located in the exon and intron regions, respectively, and the remaining, a small group of lncRNA with ploy A tail, were in the intergenic regions (Supplementary Table S2).

Based on the sex and IMF content of these 28 Duroc pigs, four pigs with extremely high- (D8, D15, D19, and D21) and four with low-IMF content (D13, D17, D18, and D26) were chosen as the two divergent groups for comparative gene expression analysis. The mean IMF contents of the two groups were 3.70 and 1.47%, respectively. According to the alignment result of RNA sequencing data with the *S. scrofa* genome, a total of 26,918 coding genes (including 1,038 novel genes) were expressed in eight individuals with extreme IMF content. Taking into account genes with FPKM value > 1 in more than four pigs, we found 18,411 and 18,950 coding genes in the high- and low-IMF groups, respectively.

### Differentially Expressed Genes Between Low- and High-IMF Groups

**FIGURE 1:**
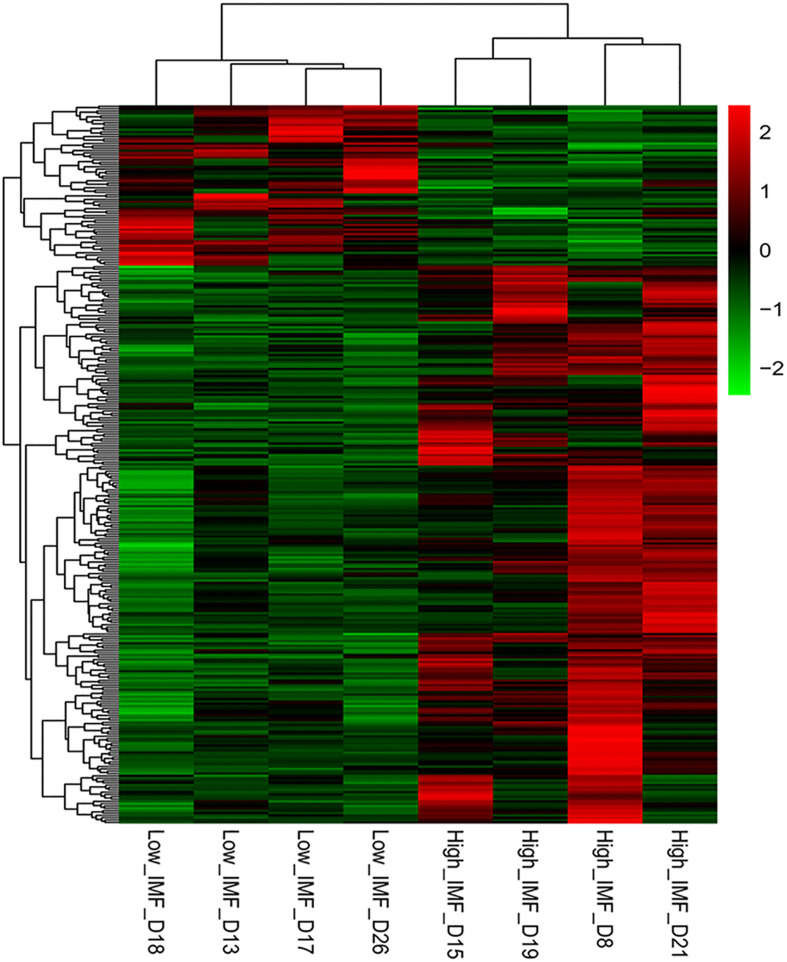
Heat map of differentially expressed genes (DEGs) between the high- and low-intramuscular fat (IMF) groups. The DEGs were detected by DEseq2 and edgeR with |log2(fold change)| ≥ 1 and *p* < 0.05.

DESeq2 and edgeR were very popular and easy-to-use packages for the differential expression analysis of RNA-seq data (Varet et al., 2016). In the present study, both methods were employed to identify DEGs between the two groups. Using the DESeq2 method in terms of |log_2_(fold change)| ≥ 1, we identified 316, 130, and 10 DEGs between the high- and low-IMF groups at *p*-value cutoffs of 0.05 and 0.01 and a *q-*value cutoff of 0.05, respectively. It suggested that the transcript differences related to IMF content between the selected pigs were small. These results were similar to those from a previous study, in which 96 and 28 DEGs related to lipid profiles were detected in term of fold-change ≥ 1.5 at cutoffs of *p* ≤ 0.01 and *q* ≤ 0.05 in Duroc pigs, respectively (Cardoso et al., 2017). On the other hand, using the edgeR packages, we detected 457, 172, and 12 DEGs between the two groups at *p*-value cutoffs of 0.05 and 0.01 and a *q* value cutoff of 0.05, respectively. A total of 309 overlapped genes detected in terms of |log_2_(fold change)| ≥ 1 and *p* < 0.05 were considered as DEGs for the following functional enrichment analyses (Supplementary Table S3). Of these, 240 and 69 genes were up- and downregulated in the high-IMF group, respectively. Figure 1 exhibits the heatmap of these DEGs, from which it can be seen that the expression patterns of DEGs are consistent within groups and different between groups.

Moreover, using DESeq2 and edgeR, only seven overlapped genes, including *SPP1*, *KCNN1*, *ENSSSCG00000034371*, *LEP*, *CIDEC*, *SFRP1*, and *ENSSSCG00000040849*, remained significant after correction for multiple testing (*q* ≤ 0.05 and |log_2_(fold change)| ≥ 1, Table 1). Among these genes, *LEP* gene encodes a protein, leptin, which is secreted by white adipocytes into the circulation and exerts physiological action through the leptin receptor (*LTPR*), which is also associated with IMF content (Roger et al., 2016; Wang et al., 2019). Consistent with our result, Gao et al. (2004) found that expression of *LEP* is significantly different in the intramuscular fat tissue between Erhualian and Large white pigs. *CIDEC* encodes an adipocyte lipid drop protein, which negatively regulates lipolysis and promotes triglyceride accumulation (Puri et al., 2007).

**TABLE 1:** Description of the top seven differentially expressed genes (DEGs) detected between high- and low-intramuscular fat (IMF) groups with |log2(fold change)| ≥ 1 and *q* < 0.05.

ID	Gene name	log_2_(fold change)	DEseq2		edgeR	
			*p*-value	*q-*value	*p-*value	*q-*value
ENSSSCG00000009216	*SPP1*	2.44	2.01E−12	6.11E−09	1.02E−08	9.69E−05
ENSSSCG00000013892	*KCNN1*	–1.73	4.37E−12	1.14E−08	1.44E−07	6.84E−04
ENSSSCG00000034371	–	3.73	1.67E−10	3.82E−07	2.14E−09	4.08E−05
ENSSSCG00000040464	*LEP*	2.00	1.16E−05	2.12E−02	4.34E−05	3.94E−02
ENSSSCG00000011557	*CIDEC*	1.83	1.16E−05	2.12E−02	2.65E−06	5.06E−03
ENSSSCG00000025822	*SFRP1*	1.37	2.24E−05	2.92E−02	5.18E−06	8.22E−03
ENSSSCG00000040849	–	–1.42	3.48E−05	4.24E−02	1.36E−05	1.72E−02

### Gene Ontology and Pathway Enrichment Analyses of DEGs

**FIGURE 2:**
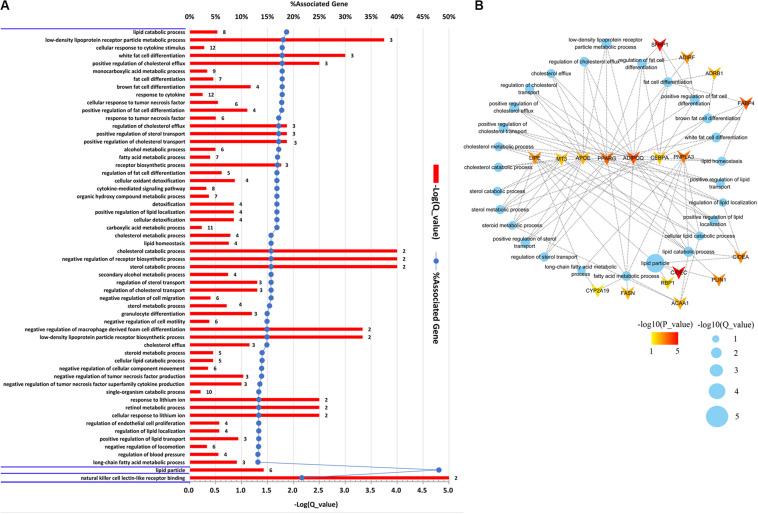
Gene Ontology (GO) enrichment analysis of differentially expressed genes (DEGs). **(A)** Significantly enriched GO terms of the DEGs detected by DEseq2 and edgeR with |log2(fold change)| ≥ 1 and *p* < 0.05. The figure is composed of three parts, biological processes, cellular components, and molecular functions, which are divided by blue horizontal lines. The significance level of enrichment was set at corrected *p*-value (*q-*value) < 0.05. **(B)** Significantly enriched GO terms related to lipid metabolism and fat cell differentiation. Blue dots represent enriched GO terms. Genes involved in GO terms are represented by V shape. The colors of genes are decided by the *p*-values examined by DEseq2.

To gain an insight into the function of DEGs detected in terms of |log2(fold change)| ≥ 1 and *p* < 0.05, we carried out GO term enrichment analysis for up- and downregulated genes, respectively. Consequentially, 58 significantly enriched GO terms (*q* < 0.05, Supplementary Table S4) were found for the upregulated genes and none for the downregulated genes. Among the 58 enriched GO terms, most belonged to the biological process (BP) category, and only two terms, lipid particle and natural killer cell lectin-like receptor binding, belonged to the molecular function (MF) and cellular component (CC) category, respectively (Figure 2A). Importantly, 27 of the 58 GO terms identified were closely associated with lipid metabolism and fat cell differentiation, such as lipid catabolic process, low-density lipoprotein receptor particle metabolic process, white fat cell differentiation, and positive regulation of cholesterol efflux (Figure 2B). Lipid particle was the most significant GO term (*q* = 1.56E−05). Moreover, other significant GO terms related to some physiological and biological events, such as response to cytokine, response to tumor necrosis factor, alcohol metabolic, and detoxification, were also enriched (Figure 2A). In addition, we conducted KEGG pathway analysis for the DEGs. Five pathways were significantly enriched for the upregulated DEGs (*q* < 0.05, Table 2), whereas none were enriched for the downregulated genes. The *q* values of the enriched pathways of DEGs showed a low level of significance. It may be one feature of muscle tissue. Similar results could be found in the previous studies (Cardoso et al., 2017; Lim et al., 2017). Of the five significant pathways, two pathways, the regulation of lipolysis in adipocytes (*q* = 5.05E−03) and the peroxisome proliferator-activated receptor (PPAR) signaling pathway (*q* = 5.05E−03), were IMF related.

**TABLE 2:** Significantly enriched pathways of differentially expressed genes (DEGs) between low- and high-intramuscular fat (IMF) groups.

Description	Gene representation	*p-*value	*q*-value	Gene name
Regulation of lipolysis in adipocytes	5/47	4.94E−05	5.05E−03	*PLIN1*, *LIPE*, *PTGER3*, *ADRB1*, *FABP4*
Retinol metabolism	4/47	1.20E−04	5.05E−03	*CYP2A19*, *SDR16C5*, *RETSAT*, *AOX1*
PPAR signaling pathway	5/47	1.34E−04	5.05E−03	*PLIN1*, *ACAA1*, *PPARG*, *ADIPOQ*, *FABP4*
Vitamin B6 metabolism	2/47	1.77E−03	4.14E−02	*PSAT1*, *AOX1*
cAMP signaling pathway	6/47	1.83E−03	4.14E−02	*LIPE*, *PTGER3*, *HCAR1*, *ADRB1*, *PPP1R1B*

The DEGs involved in GO terms and pathways related to lipid metabolism and fat cell differentiation were further analyzed. A total of 18 DEGs, as illustrated in Figure 2B, were found to be involved in the 27 significantly enriched GO terms related to IMF content. Moreover, of the 18 genes, *APOE*, *ADIPOQ*, *PPARG*, *CEBPA*, *LIPE*, *MT3*, and *PNPLA3* were involved in more than five GO terms (Figure 2B). Two IMF-related pathways covered eight genes, *PLIN1*, *LIPE*, *PTGER3*, *ADRB1*, *FABP4*, *ACAA1*, *PPARG*, and *ADIPOQ*, which were relevant to lipid metabolism and fat cell differentiation. It is noticeable that, except for *PTGER3*, all the others overlapped with the 18 upregulated DEGs covered by enriched GO terms (Figure 2B). Therefore, functional enrichment analysis of DEGs detected 19 genes that were involved in GO terms and pathways related to lipid metabolism and fat cell differentiation. These DEGs would be regarded as key genes and used in the following detection of candidate genes.

### Coexpressed Gene Modules Associated With IMF Content

**FIGURE 3:**
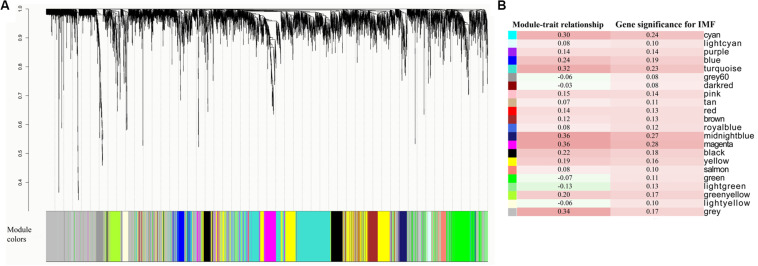
Coexpressed gene modules detected by weighted gene coexpression network analysis (WGCNA). **(A)** Cluster dendrogram showing the coexpression modules defined by WGCNA and labeled by colors. **(B)** Module–trait relationships and gene significances of each module.

The WGCNA relies on the assumption that strongly correlated expression levels of gene sets indicate that the genes work cooperatively in related pathways and contribute to the resulting phenotype. Based on this assumption, we further carried out WGCNA for all the expressed genes. By sample clustering with all genes, D22, deviating from other samples and exceeding the cutoff height, was considered to be an outlier (Supplementary Figure S1). Therefore, in WGCNA, we constructed a coexpression network with the other 27 individuals and ultimately identified 22 modules (Figure 3A). Critical modules associated with IMF content were first selected based on two criteria: MTRs > 0.3 and MS > 0.25. The results of the MTRs showed that five modules, including cyan, turquoise, gray, midnight blue, and magenta, were moderately correlated with IMF content (correlation coefficients ranging from 0.30 to 0.36, correlation *p* < 0.1, Figure 3B). The MS of the five gene modules was also detected for understanding the relationship between expression profiles and the phenotypic values based on the GS of each module genes. Among these modules, there were two modules, midnight blue (88 genes, MS = 0.27) and magenta modules (208 genes, MS = 0.28) whose MS reached 0.25 (Figure 3B). The GS of midnight blue and magenta module genes varied from −0.07 to 0.50 and from −0.28 to 0.65 with 0.11 and 0.13 of standard deviation, respectively. Consequently, based on the combined results of MTRs and MS, midnight blue and magenta modules were selected for the downstream functional enrichment analysis. The detail information about genes in the two modules is presented in Supplementary Tables S5, S6.

**FIGURE 4:**
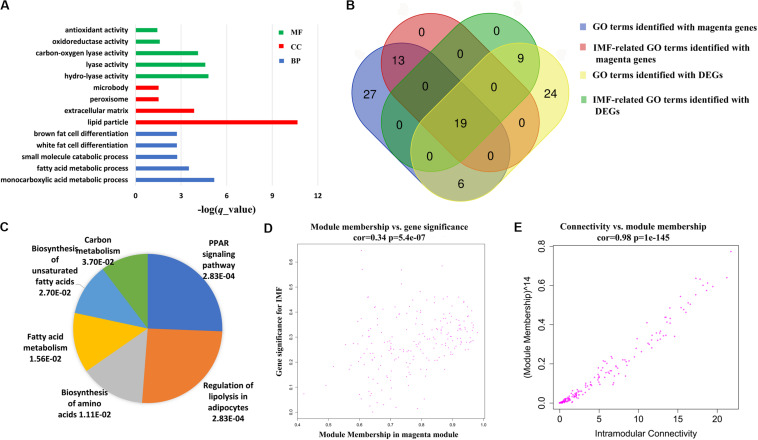
Coexpressed genes in magenta module. **(A)** The top 5 biological processes (BP) terms, all cellular components (CC), and molecular functions (MF) of Gene Ontology (GO) terms identified with magenta module genes. **(B)** Venn diagram of GO terms identified with magenta module genes and differentially expressed genes (DEGs). **(C)** Pie chart of all significant pathways (*q* < 0.05) in the magenta module. Each sector of the pie chart is proportional to the log_10_ (-*q* value) of each pathway it represents. **(D)** Association between the module membership (MM) and gene significance (GS) in the magenta module. **(E)** Association between the intramodular connectivity and module membership (MM) in the magenta module.

Further functional enrichment analyses were conducted on the genes in each module to understand the biological function of the midnight blue and magenta modules. The significant GO terms and pathways (*q* < 0.05) are presented in Supplementary Tables S7, S8. It can be seen that genes in the magenta module were significantly enriched in GO terms and pathways related to lipid metabolism (*q* < 0.05, Supplementary Table S7). Among these GO terms, 32 were IMF related, such as fat cell differentiation, fatty acid metabolic process, and lipid storage. Figure 4A illustrates the top 5 BP terms and all CC and MF terms of 65 GO terms identified with the magenta module genes. We found that there were 25 GO terms that overlapped between the GO terms identified by DEGs and magenta module genes (Figure 4B and Supplementary Table S9). In addition, 19 of them were IMF related, such as lipid metabolism, lipoprotein metabolism, cholesterol metabolism, sterol metabolism, and fat cell differentiation (Figure 4B and Table 3). This suggested that the results of the functional enrichment analysis of DEGs and magenta module genes could corroborate each other.

**TABLE 3:** Intramuscular fat (IMF)-related Gene Ontology (GO) terms overlapped between significantly enriched GO terms of differentially expressed genes (DEGs) and those of magenta module genes.

	GO terms of DEGs			GO terms of magenta module genes		
	Gene representation	*p-*value	*q-*value	Gene representation	*p-*value	*q-*value
Fatty acid metabolic process	7/71	1.78E−04	2.02E−02	14/166	2.52E−07	3.05E−04
White fat cell differentiation	3/71	3.38E−05	1.64E−02	4/166	4.68E−06	1.91E−03
Brown fat cell differentiation	4/71	7.47E−05	1.64E−02	6/166	5.00E−06	1.91E−03
Positive regulation of lipid localization	4/71	2.69E−04	2.08E−02	7/166	5.86E−06	1.91E−03
Lipid catabolic process	8/71	6.86E−06	1.35E−02	12/166	6.79E−06	1.91E−03
Regulation of lipid localization	4/71	1.23E−03	4.66E−02	8/166	1.79E−05	3.61E−03
Sterol metabolic process	4/71	5.29E−04	2.89E−02	7/166	3.44E−05	5.96E−03
Fat cell differentiation	7/71	7.44E−05	1.64E−02	10/166	5.45E−05	7.78E−03
Positive regulation of sterol transport	3/71	1.53E−04	1.92E−02	4/166	6.31E−05	8.05E−03
Positive regulation of cholesterol transport	3/71	1.53E−04	1.92E−02	4/166	6.31E−05	8.05E−03
Positive regulation of lipid transport	3/71	1.26E−03	4.66E−02	5/166	1.18E−04	1.42E−02
Regulation of fat cell differentiation	5/71	2.05E−04	2.08E−02	7/166	1.32E−04	1.46E−02
Positive regulation of fat cell differentiation	4/71	9.39E−05	1.68E−02	5/166	1.50E−04	1.52E−02
Cholesterol metabolic process	4/71	3.69E−04	2.69E−02	6/166	1.71E−04	1.56E−02
Positive regulation of cholesterol efflux	3/71	6.13E−05	1.64E−02	3/166	5.02E−04	3.05E−02
Steroid metabolic process	5/71	8.79E−04	4.02E−02	8/166	6.47E−04	3.55E−02
Regulation of sterol transport	3/71	4.68E−04	2.70E−02	4/166	8.18E−04	3.81E−02
Regulation of cholesterol transport	3/71	4.68E−04	2.70E−02	4/166	8.18E−04	3.81E−02
Lipid particle	6/67	1.65E−07	1.56E−05	12/170	1.05E−13	2.19E−11

Six significantly enriched pathways were discovered in the KEGG analysis for the genes of the magenta module (*q* < 0.05, Figure 4C). It is worth noting that regulation of lipolysis in adipocytes and PPAR signaling pathway, which were identified in the KEGG analysis of the DEGs, were also identified. According to the KEGG database^2^, regulation of lipolysis in adipocytes is a pathway regulating triacylglycerol to release fatty acids and glycerol for other organs as energy substrates. Fat deposition is a dynamic process between lipid synthesis and degradation. Some studies have demonstrated that IMF content in LD is determined by a balance between fat accumulation and degradation (Jeong et al., 2012). It is implied that more lipid storage may lead to the increase in lipolysis (Serr et al., 2013). Consequently, in this study, we found that the regulation of lipolysis in adipocytes pathway was enriched in the high-IMF group compared with that in the low-IMF group. The PPAR signaling pathway is a canonical pathway involved in lipid metabolism. Expression patterns of many candidate genes in this pathway were correlated with meat quality traits in the LD muscles of pigs (Wang et al., 2016). Furthermore, previous studies showed that the PPAR signaling pathway was a significant enriched pathway for DEGs of subcutaneous and intramuscular stromal vascular cells during adipogenic differentiation (Jiang et al., 2013) and for DEGs detected by transcriptome analysis of LD muscles between Laiwu and Yorkshire pigs (Chen et al., 2017). This has provided new evidence that DEG analysis and WGCNA could corroborate each other.

**FIGURE 5:**
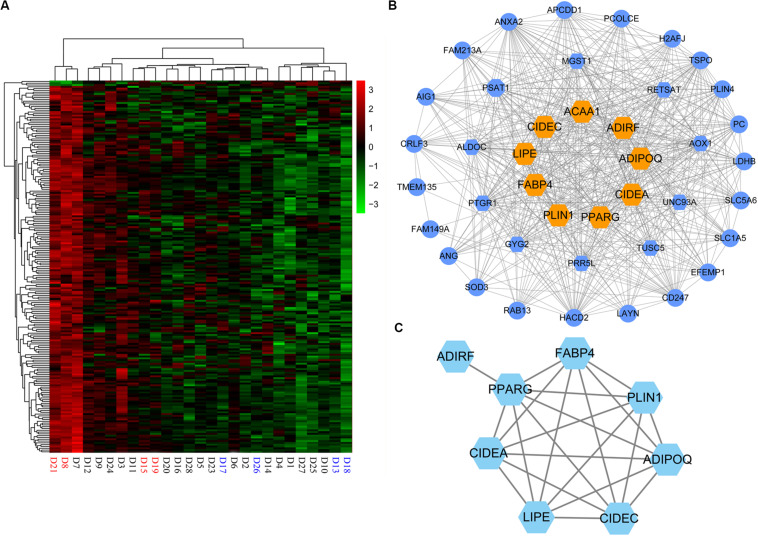
Hub genes in magenta module. **(A)** Heatmap of the magenta module genes. **(B)** Network visualization of the coexpression of 43 hub genes in magenta module. Hexagon represents 16 differentially expressed genes (DEGs) of the hub genes; orange hexagon represents nine DEGs in magenta module overlapping with 19 hub genes detected by functional enrichment analysis of DEGs. **(C)** Protein–protein interaction network of nine hub genes. ACAA1 without protein–protein interactions with the others is not displayed in the network.

In addition, for the magenta module, the correlation between MM and GS (correlation = 0.34, *p* = 5.4e−07), and the MM and connectivity (correlation = 0.98, *p* = 1e−145) both reached either moderate or strong levels (Figures 4D,E). These suggest that the magenta module is a critical module, and some of the genes in it could be candidate genes affecting the IMF content. Totally, there were 200 genes in the module. As expected, the expression of these genes varied with individuals (Figure 5A), and 39 genes out of them were overlapped with DEGs detected in terms of |log2(fold change)| ≥ 1 and *p* < 0.05.

### Detection of Candidate Genes That Affect IMF Content

As the magenta module resulting from the WGCNA showed a strong association with IMF, we investigated the candidate genes that affected IMF content in this module. First, hub genes of magenta module were identified. As hub genes for each module, their expression should first correlate significantly to module eigengene (MM > 0.85), which would suggest that the gene was a member of the magenta module. In addition, the expression of these genes should either moderately or strongly correlate to IMF value (GS > 0.3) and have more connectivity with other coexpression genes (intramodular connectivity > 5). Under these criteria, we selected 43 hub genes (Supplementary Table S10), 16 of which were DEGs, which were detected in terms of |log2(fold change)| ≥ 1 and *p* < 0.05 (Figure 5B). When comparing these 16 genes with the 19 key genes detected by the functional enrichment analysis of DEGs, we found that nine genes overlapped, viz. *ADIPOQ*, *PPARG*, *LIPE*, *CIDEC*, *PLIN1*, *CIDEA*, *ACAA1*, *ADIRF*, and *FABP4*.

Then, to evaluate the interactive relationships of the nine overlapped genes, we conducted a protein–protein interaction (PPI) network analysis using the STRING version 11.0^3^ online software. PPI networks were visualized and analyzed using Cytoscape 3.6.1 software. Except for *ACAA1* and *ADIRF*, the other seven genes, i.e., *ADIPOQ*, *PPARG*, *LIPE*, *CIDEC*, *PLIN1*, *CIDEA*, and *FABP4*, exhibited protein–protein interactions with each other (Figure 5C).

IMF content is determined by the number (hyperplasia) and size (hypertrophia) of adipocytes within the muscle (Zhao and Gao, 2009). The differentiation of intramuscular preadipocytes into intramuscular adipocytes starts during the embryonic growth and continues immediately after birth, then slow down during the growth of the animal (Sepe et al., 2011). The hypertrophy process is determined by the ratio between lipogenesis and lipolysis in mature adipocytes. Some previous studies have proved these seven genes as relevant candidate genes because of their important roles in these biological processes related to IMF content. Adiponectin, encoded by the *ADIPOQ* genes, is a protein hormone secreted by adipocytes involved in the regulation and inhibition of lipogenesis and the stimulation of fatty acid oxidation (Yuchang et al., 2005). Expression of *ADIPOQ* was higher in Lantang, a high-IMF pig breed, compared to that in Landrace, a low-IMF pig breed (Kaifan et al., 2013). Hormone-sensitive lipase gene (*LIPE*) is one of the most important factors in controlling lipolysis and fat accumulation in animals (Guenter et al., 2003). *LIPE* were found to be significantly higher expressed in pigs with low-IMF content (Zappaterra et al., 2016), its polymorphisms associated with pig IMF content (Xue et al., 2015), and, in the candidate regions under positive selection of Laiwu pigs, one Chinese indigenous pig breed with extremely high proportion of IMF content (Chen et al., 2017). *CIDEA* and *CIDEC* are two members of the novel CIDE family of apoptosis-inducing factors. The expression of *CIDEA*, an adipose-specific gene, was associated with the terminal differentiation of fat cells (Danesch et al., 1992). It was discovered that both *CIDEA* and *CIDEC* were highly expressed in adipose tissues, and their expression levels were significantly higher in obese pigs than in their lean counterparts (Li et al., 2009). Notably, *CIDEC* was one of the top 7 DEGs distinguishing at |log2(fold change)| value ≥ 1 and *q* < 0.05 (Table 1).

The genes, *PPARG*, *PLIN1*, and *FABP4*, all belong to the significantly enriched PPAR signaling pathway, and *PPARG* was the core regulator gene of this pathway. *PPARG* regulates lipid metabolism and glucose homeostasis and promotes adipocyte differentiation and fat deposition (Vanden Heuvel and Peters, 2010). Previous studies have found that the polymorphisms of *PPARG* could significantly affect gene expression and intramuscular fat deposition in pigs (Wang et al., 2013). In addition, *PPARG* could enhance *PLIN1* expression by DNA demethylation on PPAR-response elements of *PLIN1* gene promoter upon differentiation (Fujiki et al., 2013). PLIN1 protein plays a key role in the regulation of the extra-myocellular lipid storage in pigs, which mainly determines the IMF content (Gandolfi et al., 2011). *PLIN1* was detected as a key gene affecting porcine IMF based on transcriptome and knockdown analysis (Li et al., 2018). Furthermore, once activated, the PPARG complex can recruit other transcription factors and activate the adipogenic gene transcription by the PPAR responsive elements including *FABP4* (Yutaka et al., 2008). As an adipokine, *FABP4* is secreted from adipocytes and induced during adipocyte differentiation. In pigs, *FABP4* levels were associated with the number of adipocytes and IMF content (Damon et al., 2006). Gerbens et al. (1998) found that a microsatellite sequence in the first intron of porcine *FABP4* is associated with IMF content in Duroc population, and approximately 1% IMF was observed between certain genotype classes. In bovine, polymorphisms in *FABP4* associated with IMF content were also found (Bartoň et al., 2016). Furthermore, differing from adipogenic differentiation of muscle stem cells, a novel mechanism for fatty infiltration, which might be regulated by inhibition of *FABP4*, was found in mouse (Lee et al., 2017).

To sum up, according to the results of the DEG analysis, WGCNA, and the relevant literature, the seven genes discussed above, *ADIPOQ*, *PPARG*, *LIPE*, *CIDEC*, *PLIN1*, *CIDEA*, and *FABP4*, could be potential candidate genes affecting IMF content in pigs.

## Conclusion

In this study, we performed a high throughput RNA sequencing to evaluate the transcriptome profiles differences of eight Duroc pigs with extreme IMF content. A total of 309 DEGs were screened using the DESeq2 and edgeR packages. The GO terms and pathway enrichment analysis of DEGs and WGCNA of 28 Duroc pigs revealed some potential candidate genes, such as *ADIPOQ*, *PPARG*, *LIPE*, *CIDEC*, *PLIN1*, *CIDEA*, and *FABP4*, and pathways, such as regulation of lipolysis in adipocytes and PPAR signaling pathway. These potential candidate genes and pathways play an essential role in affecting the IMF content of pigs, and further studies should be carried out to unravel their specific mechanism on IMF content.

## Data Availability Statement

All datasets generated for this study are included in the article/Supplementary Material. The raw data of RNA sequencing have been deposited in the National Center for Biotechnology Information Sequence Read Archive with accession no. PRJNA527944 (available online: https://www.ncbi.nlm.nih.gov/sra/PRJNA527944).

## Ethics Statement

The animal study was reviewed and approved by the Institutional Animal Care and Use Committee of Institute of Animal Husbandry and Veterinary Medicine, Shandong Academy of Agricultural Sciences (approval code, IACC20060101, 1 January 2006).

## Author Contributions

JW conceived and designed the experiments and explained the data. XZ analyzed the main content of the data with the help of HH and HL. YW collected the samples and examined the phenotype with the help of CW and HL. XZ wrote the manuscript with the help of JW. All authors contributed to the article and approved the submitted version.

## Conflict of Interest

The authors declare that the research was conducted in the absence of any commercial or financial relationships that could be construed as a potential conflict of interest.

## Footnotes

1 https://www.animalgenome.org/cgi-bin/QTLdb/SS/index

2 https://www.genome.jp/dbget-bin/www_bget?map04923

3 https://string-db.org

## Supplementary Material

FIGURE S1 Sample clustering with all expressed genes to detect outliers.

TABLE S1 Phenotypic information of 28 Duroc pigs.

TABLE S2 Summary of sequencing data of 28 Duroc pigs.

TABLE S3 DEGs detected by DESeq2 and edgeR between high- and low-IMF groups (|log2(foldchange)| ≥ 1, *p* < 0.05).

TABLE S4 Significantly enriched GO terms (*q* < 0.05) of the DEGs detected between high- and low-IMF groups.

TABLE S5 Genes in the magenta module.

TABLE S6 Genes in the midnight blue module.

TABLE S7 Significantly enriched GO terms and pathways (*q* < 0.05) of the magenta module genes.

TABLE S8 Significantly enriched GO terms and pathways (*q* < 0.05) of genes in the midnight blue module.

TABLE S9 Overlapped GO terms identified by DEGs and magenta module genes.

TABLE S10 Description of the 43 hub genes in the magenta module.
