# Supplementary figures and images for: Muscle Transcriptome Analysis Reveals Potential Candidate Genes and Pathways Affecting Intramuscular Fat Content in Pigs
Source: Front Genet. 2020 Aug 11;11:877. doi: 10.3389/fgene.2020.00877 (PMC7431984; doi:10.3389/fgene.2020.00877)

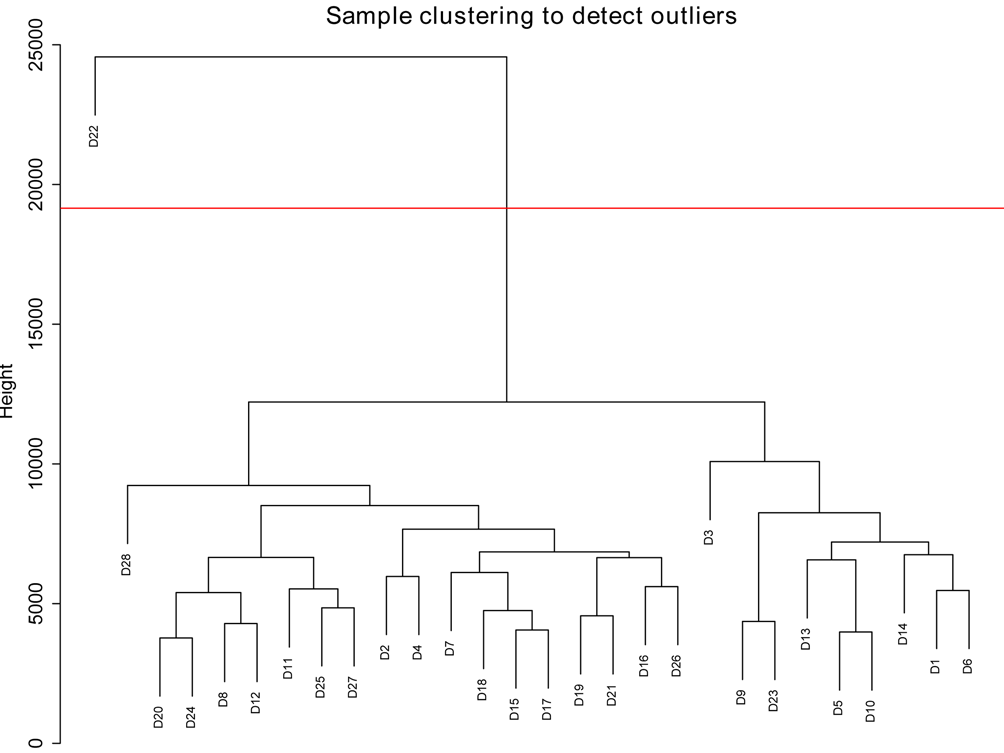

Supplement: FIGURE S1 — Sample clustering with all expressed genes to detect outliers. [file Image_1.TIF]
